# Environmental Strategies of Affect Regulation and Their Associations With Subjective Well-Being

**DOI:** 10.3389/fpsyg.2018.00562

**Published:** 2018-04-18

**Authors:** Kalevi M. Korpela, Tytti Pasanen, Veera Repo, Terry Hartig, Henk Staats, Michael Mason, Susana Alves, Ferdinando Fornara, Tony Marks, Sunil Saini, Massimiliano Scopelliti, Ana L. Soares, Ulrika K. Stigsdotter, Catharine Ward Thompson

**Affiliations:** ^1^Faculty of Social Sciences, Psychology, University of Tampere, Tampere, Finland; ^2^Institute for Housing and Urban Research, Uppsala University, Uppsala, Sweden; ^3^Institute of Psychology, Leiden University, Leiden, Netherlands; ^4^Center for Behavioral Health Research, College of Social Work, University of Tennessee, Knoxville, Knoxville, TN, United States; ^5^Department of Architecture, Çankaya University, Ankara, Turkey; ^6^Department of Psychology, University of Cagliari, Cagliari, Italy; ^7^School of Behavioural, Cognitive and Social Sciences, University of New England, Armidale, NSW, Australia; ^8^Indian Association of Health, Research and Welfare, Hisar, India; ^9^Department of Human Studies, Libera Università Maria Ss. Assunta, Rome, Italy; ^10^Instituto Superior de Agronomia, Universidade Técnica de Lisboa, Lisbon, Portugal; ^11^Department of Geosciences and Natural Resource Management, University of Copenhagen, Copenhagen, Denmark; ^12^OPENspace Research Centre, University of Edinburgh, Edinburgh, United Kingdom

**Keywords:** affect regulation, natural places, urban places, perceived efficacy, life satisfaction, perceived health, emotional well-being, coping strategy

## Abstract

Environmental strategies of affect regulation refer to the use of natural and urban socio-physical settings in the service of regulation. We investigated the perceived use and efficacy of environmental strategies for regulation of general affect and sadness, considering them in relation to other affect regulation strategies and to subjective well-being. Participants from Australia, Finland, Germany, Great Britain, Italy, India, the Netherlands, Portugal, and Sweden (*N* = 507) evaluated the frequency of use and perceived efficacy of affect regulation strategies using a modified version of the Measure of Affect Regulation Styles (MARS). The internet survey also included the Satisfaction with Life Scale (SWLS), emotional well-being items from the RAND 36-Item Health Survey, and a single-item measure of perceived general health. Environmental regulation formed a separate factor of affect regulation in the exploratory structural equation models (ESEM). Although no relations of environmental strategies with emotional well-being were found, both the perceived frequency of use and efficacy of environmental strategies were positively related to perceived health. Moreover, the perceived efficacy of environmental strategies was positively related to life satisfaction in regulating sadness. The results encourage more explicit treatment of environmental strategies in research on affect regulation.

## Introduction

It is striking that so many comprehensive reviews, inventories and categorizations of affect regulation strategies (including coping methods) have ignored the use of environmental strategies, that is, the use of specific socio-physical settings and their experiential contents as a common means of affect and stress regulation (Skinner et al., 2003; Folkman and Moskowitz, 2004; Larsen and Prizmic, 2004; Taylor and Stanton, 2007; Naragon-Gainey et al., 2017). Conceptually, affect regulation includes emotion- and mood-regulation and refers to the modulation of feeling states, including the valence and the energy level of those states (Larsen and Prizmic, 2004). When the focus is on down-regulation of negative affect, affect regulation is analogous to stress-regulation and coping. Especially emotion-focused coping strategies may overlap with mood-regulation concepts and strategies (Larsen, 2000). Research in affect regulation has predominantly focused on covert emotion regulation strategies occurring within the individual rather than on the examination of overt, behavioral emotion regulation strategies (Aldao and Dixon-Gordon, 2014), leaving the role of socio-physical settings underexplored.

Several commonly used strategies to down-regulate negative emotions have been clustered in three main categories: disengagement (distraction, behavioral avoidance, experiential avoidance, expressive suppression), aversive cognitive perseveration (worry, rumination, experiential avoidance), and adaptive engagement (problem solving, reappraisal, mindfulness, and acceptance; Naragon-Gainey et al., 2017). Coping categorizations (including affect regulation) have usually been clustered as problem-focused, emotion-focused, meaning-focused, and social coping (Folkman and Moskowitz, 2004). Strategies like avoidance, escape, distraction, isolation, or social withdrawal all potentially implicate escape or withdrawal from unsupportive environments and contexts (Skinner et al., 2003), but the places *to where* people escape have remained uninvestigated. Research on environmental affect regulation or environmental coping is glaringly lacking. This lacuna has persisted despite the fact that early definitions of emotion- and self-regulation included interaction with the physical environment (Campos et al., 1989; Dodge and Garber, 1991). Moreover, the role of the environment has been fundamentally acknowledged in the phenomena of niche-picking (Campos et al., 2004) and niche-building (Tesser, 2002), in which a person avoids settings or situations where undesired emotions may become activated and chooses or creates settings or situations where desired emotions are likely. Similarly, certain process models of emotion regulation include “antecedent focused regulation” which includes situation selection and situation/environment modification, in which a person approaches, avoids, or modifies situations or environments on the basis of their likely emotional impact (Gross, 1998).

As explicit environmental strategies are lacking in the current inventories and categorizations of affect regulation, we aim to start filling this gap by including such a strategy in an existing affect regulation inventory and investigating its relationships with other affect regulation strategies and with subjective well-being. With environmental strategies we refer to the use of specific places or socio-physical settings in the service of affect regulation, such as visiting a nearby favorite place in a natural or urban setting to calm down (Korpela, 1992). In the following, we begin by reviewing literature speaking to the necessity of recognizing the importance of environmental strategies of affect regulation. We first consider work in diverse areas of psychology that only implicitly acknowledges the use of environmental strategies. We then consider work done in environmental psychology and allied disciplines that addresses these strategies explicitly. Last, we describe the relationship between affect regulation and well-being.

### Implicit treatment of relations between affect regulation and socio-physical settings

Outside of environmental psychology, few psychological studies have explicitly related affect regulation strategies to specific places or socio-physical settings. Rather, the treatment of environment has been general or implicit. For example, it has been reported that strategies of changing a bad mood and raising energy and alertness include such behaviors as “changing location” and “going out to get some fresh air” (Thayer et al., 1994). In an attempt to conceptually capture the diversity of affect-regulation strategies, Parkinson and Totterdell (1999) searched within several empirical studies and produced a list of 162 relatively distinct strategies. “Go to a favorite place” was included in the behavioral cluster of these strategies, but the authors did not comment on this finding.

Assuming that going to a favorite place is included in a category of “a pleasant or relaxing activity” as was the case in Parkinson and Totterdell's (1999) study, a prospective, time-sampling field study of trainee teachers further strengthened the view that favorite place visits are quite important in affect regulation (Totterdell and Parkinson, 1999). Namely, trainee teachers spontaneously used diversion methods (pleasant or relaxing activities, active or energetic activities, cognitive distraction and avoidance) more than engagement methods (rationalization, reappraisal, social support, venting) in a study where the participants reported on their mood and mood-regulation strategies at regular intervals (every 2nd hour) over 2 weeks. Cognitive distraction was used most often (62%), while pleasant or relaxing activities ranked third (53%). Moreover, pleasant or relaxing activities were the most powerful predictors of cheerfulness and calmness. Thus, the trainee teachers were most successful in improving their moods when they did something distracting, pleasant or relaxing in particular. As mentioned, it is appropriate to assume that these activities involve visiting favorite or at least well-liked environments. This assumption is further encouraged by a study in which pleasant activities, including looking at the sky or clouds, seeing beautiful scenery, taking a walk, traveling, and breathing clean air, were correlated with mood over a 30-day period in student groups differing in mental health status (Lewinsohn and Libet, 1972).

In an exploratory study using semi-structured interviews with adjudicated and non-adjudicated adolescents and their parents (*N* = 20), it was found that adolescents relied heavily on leaving the scene, such as going for walks or to their room and engaging in distracting activities as a means of socially regulating their affect and managing anger (Keiley and Seery, 2001). In all, looking across the literature from much of the discipline of psychology, one finds few studies that refer directly to the use of places and settings in affect regulation, and they say little about the specific places that people go to for affect regulation or the incidence and relative effectiveness of such regulation strategies.

### Environmental psychological studies relating socio-physical settings to affect regulation

Research in environmental psychology and allied environmental professions like landscape architecture provides ample evidence of the use of specific settings in affect regulation. A strong theme in this literature concerns visits to natural settings (e.g., beach, forest, park) as frequently-used and effective strategies in the regulation of stressful affects (Korpela, 1992; Sampson and Gifford, 2010; Ward Thompson and Aspinall, 2011). For example, one study simply asked university students to write about places they took their problems to feel better (Francis and Cooper, 1991). These were most often natural, park-like places. One third described their bad mood getting better, one third reported getting perspective on their worries, and one fourth reported diversion of thoughts. Only one of every seven reported no improvement in mood. Another study surveyed visitors to four metropolitan parks (Hammitt, 2000). Important for the present study, it was found that being away-from and being away-to were distinct concepts in the minds of visitors. Being away-to nature, associated with quiet, privacy, and coping with everyday hassles was more important than being away-from crowded urban places and everyday routines. In another survey study, 91% of a probability sample of Norwegian adults endorsed at least one of three statements about the use of natural settings for affect regulation (“I need time in nature to be happy”; “Sometimes when I am unhappy I find comfort in nature”; “Being out in nature is a great stress reducer for me”), and 65% endorsed all three statements (Hartig et al., 2007).

Experimental evidence affirms that visiting or seeing natural environments—typically parks or woodlands—is relatively effective in alleviating both mental fatigue and emotional stress, at least in comparison to the outdoor urban spaces that may otherwise be available for escape (e.g., Parsons et al., 1998; Hartig et al., 2003; Berman et al., 2008; for a review, see Ohly et al., 2016). This alleviation, called *restoration*, can involve diverse outcomes, including renewal of a capacity to direct attention, physiological changes from tension and stress toward relaxation, and positive mood change.

Some of the evidence concerning the use of natural settings in affect regulation comes from studies of settings that people identify as their favorite place. Whether referring to a natural setting or not, accounts concerning favorite places commonly carry themes of affect regulation (Korpela et al., 2001). For example, cross-sectional self-report studies indicate that people recognize that favorite nearby places provide restorative, stress-alleviating experiences such as relaxation, a decrease in negative feelings and an increase in positive ones, and that people visit these places for the regulation of their self-experience and feelings (Newell, 1997; Gulwadi, 2006; Mason et al., 2010; Johnsen, 2013). In a controlled field experiment, subjects who visited nearby favorite places experienced significantly stronger stress-alleviation (e.g., calmness, attentiveness, relaxation) than subjects in non-visiting and control groups during 5 days (Korpela and Ylén, 2009). Although restorative outcomes such as relaxation, positive mood and self-reported attentional recovery can occur in many different favorite places, they have been reported to be more intense in favorite natural settings than in favorite urban settings (Korpela et al., 2010).

In summary, visiting socio-physical environments, particularly natural settings that may be favorite ones, serves affect regulation, regulation of attentional fatigue, and stress reduction. Despite explicit efforts to connect environmental stress and restorative environments research (Hartig and Staats, 2003), favorite place and restorative environment studies have not yet contributed to the instruments used to measure coping responses and affect regulation strategies. In research in areas such as stress and coping, recovery from work, and leisure experience, there is great potential for inventorying and comparing regulation methods with environmental regulation methods to estimate their relative perceived frequency of use, efficacy and judged importance.

### Affect regulation strategies and well-being

We maintain that visiting or seeing natural environments and favorite places alleviates both post-visit attentional fatigue and stress and thereby affects other aspects of well-being. For example, nature exposure increases positive affect with a larger effect size than it decreases negative affect (McMahan and Estes, 2015). Good perceived health has been associated with proximity to the nearest green space (Stigsdotter et al., 2010), mediated by recreational walking (Sugiyama et al., 2008). More green space in residential areas has been associated with lower levels of depression in a twin-study design (Cohen-Cline et al., 2015). Moreover, moving to greener areas has been related with greater subsequent happiness and life satisfaction over several years (Alcock et al., 2014), suggesting long-term effects of the use of nearby environments for emotional well-being and life satisfaction.

Within the stress and coping literature, approach-oriented coping strategies, such as problem-directed action, have been tied to positive psychological and physical health outcomes in stressful circumstances (Taylor and Stanton, 2007). Although the avoidance coping strategies, such as withdrawal and distraction, can be successful for coping with short-term uncontrollable stressors, generally they are related to increased distress and even mortality (Taylor and Stanton, 2007). For example, reliance on the avoidant coping strategies has been found to be associated with higher levels of anxiety in students (Park and Adler, 2003) and depressive symptoms in late-middle-aged people (Holahan et al., 2005).

Studies suggest that emotion-focused strategies, such as engaging in pleasant activities, withdrawing from the situation, and venting or suppressing one's emotions, might be useful in situations where individuals do not have the ability to change the situation causing negative consequences (Bonanno et al., 1995). For example, sadness is an emotion that often includes feelings of hopelessness and causes people to withdraw and isolate (Shaver et al., 1987). As people withdraw to somewhere (i.e., to a socio-physical setting) seeking emotional relief, in this study we pursue the role of the environment not only in affect regulation in general but also in sadness regulation more specifically (cf. Hartig, 2005).

Presumably, environmental affect regulation involves both avoidance- and approach- focused coping strategies. Moreover, this may occur in a particular temporal sequence; a person may withdraw to a specific socio-physical setting to support restoration, thus enabling more effective instrumental coping with the given problem at a later time. Much further study is needed in this field, starting with a clearer understanding of the variety and use of environmental affect regulation strategies and their correlation with other kinds of strategies.

### Study aims

In all, the main purpose of this study is to investigate the perceived use and effectiveness of environmental affect regulation strategies in relation to other regulation strategies. In that endeavor, we use an existing affect regulation measurement instrument and complement it with items representing environmental strategies to see whether these items form a distinct factor. We expect that people recognize environmental regulation as a separate set of strategies of affect regulation, for the regulation of feelings in general (*Hypothesis 1a*) and for sadness in particular (*Hypothesis 1b*). We acknowledge the challenge of empirically separating different strategies when in practice factors for different strategies may be correlated because they often occur together or they are combined in a particular temporal sequence. For example, an environmental strategy may be intended to facilitate some other strategy, as when a person goes for a walk in a pleasant park to withdraw from an unpleasant situation for cognitive reappraisal and to put things in perspective. Investigating such sequences, however, is beyond the scope of the present study.

As the goal of affect regulation is to increase subjective well-being, the relationships between environmental and other affect regulation strategies and subjective well-being are also examined. We use Diener's (2000) definition of subjective well-being and include the major aspects, that is, a global judgment of life satisfaction and emotional well-being representing positive affect and low levels of negative affect. We exclude satisfaction with separate life domains (e.g., work satisfaction) and include perceived general health instead (Diener, 2000), because it has been shown to have a positive relationship with nature exposure. Thus, if the set of environmental means forms an independent strategy of affect regulation in people's minds, we expect its use and efficacy to be positively associated with well-being (*Hypothesis 2*).

## Methods

### Design and procedure

We conducted two cross-sectional surveys, one for general affect regulation and one for sadness regulation. Data were gathered from different countries to ensure the heterogeneity of the sample. The majority of respondents were recruited during lectures or via e-mail lists for students. The participants were informed that the study was about “influencing feelings and well-being” and about anonymity and confidentiality in data handling. Voluntary participants filled in an internet-based questionnaire. The participants thus implicitly gave their informed consent by filling in the questionnaire. Each questionnaire took about 15 min to complete. For background information the respondents were asked to state their age, nationality, the country of residence, occupation and average income per year. The questionnaires contained a measure of affect regulation strategies and measures of subjective well-being and health. The participants received no credit or monetary compensation for their participation.

### Participants

Table 1 presents descriptive statistics for the participants of the two separate samples for the two internet-based questionnaires by the country of residence, gender and age. A total of 507 participants (372 women and 135 men) from Australia, Finland, Germany, Great Britain, Italy, India, the Netherlands, Portugal, and Sweden completed the questionnaire inquiring about the regulation of feelings in general. For the second questionnaire, concerned with the regulation of sadness, the total number of participants was 626 (464 women and 162 men). Those respondents came from Australia, Denmark, Finland, Germany, Great Britain, Italy, the Netherlands, Sweden, and the USA. A majority of the respondents were students, aged under 25 and with a low to average income level.

**Table 1:** Descriptive statistics for the respective national samples of respondents for the general affect and sadness regulation questionnaires.

	**General affect regulation**				**Sadness regulation**			
	**Total**	**Women**	**<25 years**	**Age range**	**Total**	**Women**	**<25 years**	**Age range**
	**N**	**N**	**N**		**N**	**N**	**N**	
	**%**	**%**	**%**	**years**	**%**	**%**	**%**	**years**
Australia	85	81	33	17–57	56	47	24	17–60
	16.8	95.3	38.8		8.9	83.9	42.9	
Finland	54	43	25	19–33	128	112	99	19–54
	10.7	79.6	46.3		20.4	87.5	77.3	
Germany	32	26	26	18–52	34	32	30	16–44
	6.3	81.2	81.2		5.4	94.1	88.2	
Italy	51	28	32	18–34	81	37	38	19–56
	10.1	54.9	62.7		12.9	45.7	46.9	
Netherlands	73	57	63	18–34	69	56	68	17–29
	14.4	79.1	86.1		11.0	81.2	98.6	
Sweden	84	58	51	19–50	147	98	96	19–45
	16.6	69.0	60.7		23.5	66.7	65.3	
Great Britain	40	33	21	19–57	35	30	31	18–43
	7.9	82.5	52.5		5.6	85.7	88.6	
India	66	30	50	18–30				
	13.0	45.5	75.8					
Portugal	22	16	19	20–39				
	4.3	72.7	86.4					
USA					50	33	46	19–38
					8.0	66.0	92.0	
Denmark					26	19	12	21–45
					4.2	73.1	46.2	
Total sample	507	372	320	17–57	626	464	444	16–60
	100	73.4	63.0		100	74.1	71.0	

### Measures

#### Affect regulation strategies

The data were collected using a modified version of MARS (Measure of Affect Regulation Styles; Larsen and Prizmic, 2004) (including 13 strategies) which was developed on the basis of an act-frequency study by Larsen (2000). Previous studies have used a shorter version (including 11 strategies) showing relationships of pleasant activity, venting, spending time alone and with others to negative affect, self-criticism and dependency in a sample of women (Fichman et al., 1999). However, the measure remains at an early phase of development without extensive, published validation studies. The version of the MARS used in this study includes 32 items that can be conceptually divided into 13 strategies of affect regulation. A taxonomy of these strategies is based on two orthogonal dimensions: cognitive vs. behavioral and having focus on the situation vs. on mood (Larsen, 2000). These strategies (with the examples of each) are as follows: (1) distraction, getting one's mind off negative events or emotions, avoiding rumination (I watched TV, read a book, etc., for distraction); (2) venting, expressing the negative affect, catharsis (I let my feelings out by venting or expressing them); (3) suppression, keeping the negative affect from being expressed (I tried to not let my feelings show, to suppress any expression); (4) cognitive reappraisal, finding meaning in negative events (I tried to find something good in the situation); (5) downward social comparison (I compared myself with people who are worse off); (6) problem-directed action or planning to avoid problems in the future (I took action to solve the problem causing my mood); (7) self-reward, thinking about or doing pleasant activities (I did something fun, something I really enjoy); (8) exercise, relaxation, eating, and other physical manipulations (I played sports, exercised); (9) socializing, seeking comfort, help, or advice from others (I talked to an advisor or mentor); (10) withdrawal, isolation, spending time alone (I kept to myself, I wanted to be alone); (11) gratitude, counting one's blessings, or focusing on areas of life that are going well (I tried to think about those things that are going well for me); (12) helping others, committing acts of kindness (I went out of my way to help someone); and (13) humor, laughter, expressing positive emotions (I laughed, joked around, tried to make myself or others laugh).

Based on earlier studies on favorite places (Korpela and Ylén, 2007), four items representing environmental strategies were created and scattered among the other 32 MARS items for the purposes of the present study. Two of the items were related to regulation in natural environments and two to regulation in urban environments. One of the natural environment items and one of the urban environment items was specifically about affect regulation in a favorite place. The additional statements were: “I went to my favorite place in nature,” “I went for a walk in the forest, in a park, on the beach or some other natural setting,” “I went to my favorite place in an urban setting,” and “I took a walk downtown.”

In the version of the MARS questionnaire used for general affect regulation, the respondents were asked to indicate “how frequently they use each behavior to influence their feelings, either to increase positive moods or to decrease negative moods.” In the version used for sadness regulation, they were asked to indicate “how frequently they use each behavior to influence their feelings of sadness.” They responded using a scale ranging from 0 (not at all; 1 = hardly ever; 2 = sometimes; 3 = moderate amount; 4 = often; 5 = very often) to 6 (almost always). In this study, because frequently used strategies may not always be the most effective (due to e.g., physical constraints in using the most effective strategies), the respondents were also asked to evaluate the efficacy of each behavior using the same scale (i.e., “how effective each behavior is in influencing your feelings / sadness”; 0 = not at all effective; 6 = almost always effective).

#### Subjective well-being

In addition to the background information and MARS, the respondents were asked to answer questions concerning different aspects of subjective well-being. Satisfaction with life (SWL) was measured using the Satisfaction With Life Scale (SWLS; Diener et al., 1985). The respondent is asked to indicate his/her agreement with five statements (e.g., “the conditions of my life are excellent”) using a 7-point scale (1 = strongly disagree, 7 = strongly agree). The SWLS has been shown to be a valid and reliable measure of life satisfaction (Diener et al., 1985; Pavot and Diener, 1993).

Emotional well-being (during the past 4 weeks) was measured using the emotional well-being scale from the RAND 36-Item Health Survey (Aalto et al., 1995). The scale's five items included questions about being nervous (“How much of the time during the past 4 weeks have you been a very nervous person?”), down in the dumps, peaceful, blue/sad, and happy. The participants rated the items with a 6-point scale (1 = all the time, 6 = none of the time). Responses to the negatively-phrased items were inverted for the analyses. Studies support the reliability and validity of the RAND Health Survey and its component scales (Bullinger, 1995; Sullivan et al., 1995; Hays and Morales, 2001).

Perceived general health was measured by a widely-used single question “How is your health at the moment?” with response alternatives ranging from 1 (poor) to 5 (excellent) (Bronzaft et al., 1998). Self-rated health is reported to be a valid summary of more detailed measures of health status (Bailis et al., 2003), and to correspond well with longevity (Jylhä, 2009).

#### Data analysis

The data were analyzed with exploratory structural equation modeling (ESEM) where the MARS items were defined as exploratory latent factors and the well-being outcomes as confirmatory latent factors. The exploratory approach for the MARS items was necessary as the construct validity and reliability of the scale are not extensively verified (Larsen and Prizmic, 2004). The exploratory approach also was in line with the understanding that environmental affect regulation could correlate with multiple other types of strategies as movement into a different environment would be a step in a process that also recruited other strategies. Based on previous, unpublished studies that have found 6 or 7 factors with some newly added items and without some of the original items (Larsen and Prizmic, 2006; Prizmic et al., 2011; Prizmic and Larsen, 2012) and the conventional eigenvalue criterion (>1), we started by specifying nine factors in each dataset. The criteria for retaining the exploratory factor solution were that each factor had ≥3 items loading on it (with a loading >0.32), and that each item had a communality >0.20 (Tabachnick and Fidell, 2007). If all items had sufficient communalities but not all factors had enough items loading on them, a solution with one less factor was specified. Based on previous studies, the minimum number of factors in a tested solution was 6. If the items' loading/factor criteria were not met but all variables had large enough communalities, variables with no loadings >0.32 in the 9-factor solution were dropped and the analyses, starting from nine factors, was re-run.

All analyses were conducted with Mplus version 7.4, apart from eigenvalues and ANOVA that were obtained from SPSS 23.0. As all the items were measured on ordinal 5 or 7 -point scales, they were specified as ordinal categorical in the analyses and the robust weighted least squares (WLSMV) estimator was used (Muthén and Muthén, 1998-2012). The ESEM approach allowed both confirmatory and exploratory latent factor specification. The latter were obtained by geomin rotation, suitable for the analysis of new item structures (Asparouhov and Muthén, 2009). Many affect regulation strategies may connect in spatial and temporal sequences and thus, an oblique rotation (with ε = 0.01, default for >3 factors) was used. To account for potential cultural differences between the countries, the analysis type was specified as complex which adjusts the standard errors of the estimates for non-independence of the residuals within countries (Muthén and Muthén, 1998-2012). This is however not itself an analysis of between-country differences, and no such analysis is attempted here given the small numbers of respondents from some countries.

**Figure 1:**
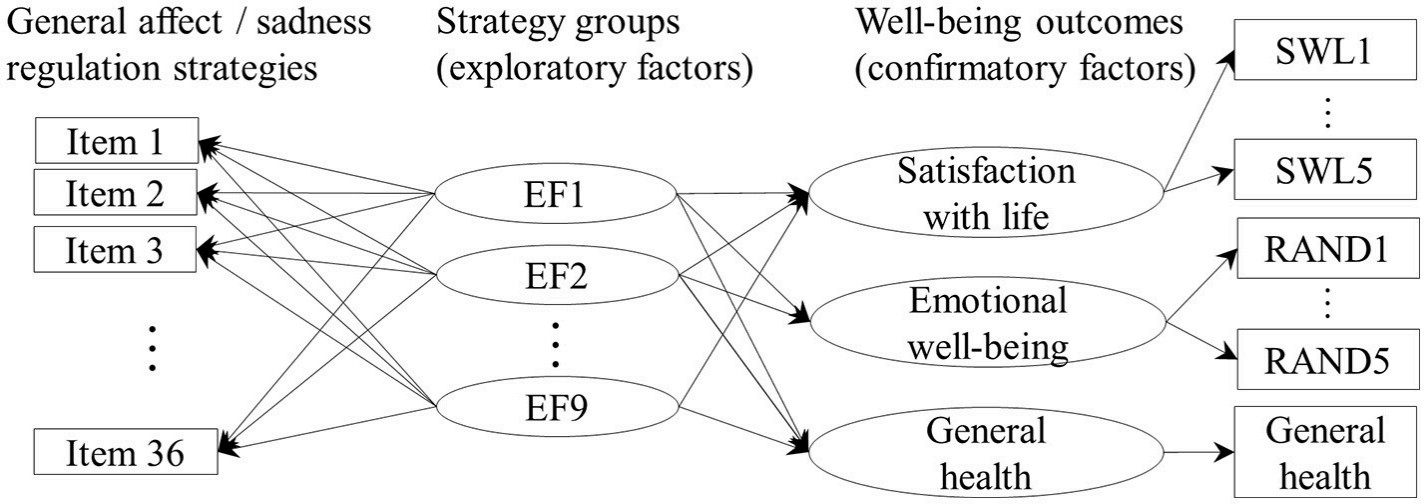
A diagram of the ESEM models showing the potential relationships assessed. For clarity, the arrows indicating residual variance of the factor indicators and the correlations between the factors are not shown.

With ESEM, the regressions of the three well-being outcomes on the exploratory MARS factors were conducted simultaneously with the exploratory factor analysis (Figure 1). Satisfaction with life and emotional well-being were specified as confirmatory latent factors with the first items being the reference items (loading fixed at 1), and general health was specified as a single-indicator ordinal variable. The analyses were conducted in the same way for the affect regulation strategies of (1) general affect, frequencies of use, (2) general affect, perceived efficacy, (3) sadness, frequencies of use, and (4) sadness, perceived efficacy. In all models, both the confirmatory outcome factors and the exploratory affect regulation factors were allowed to correlate. The model fits were assessed according to Kline's (2015) recommendations: the significance of the χ^2^ test, the pattern/size of the correlation residuals, Root Mean Square Error of Approximation (RMSEA), and Bentler Comparative Fit Index (CFI).

To evaluate the mean level of frequency of use and efficacy of the environmental strategies in relation to others, we used mean summary scores (Appendix A) of the appropriate factors in repeated measures ANOVA. We used Greenhouse-Geisser correction when the assumption of sphericity was violated.

## Results

### Factor solutions

Concerning frequencies of general affect regulation strategies, three items were dropped due to low communalities and two due to low loadings for all factors: “I drank coffee or caffeinated beverages,” “I slept or took a nap,” “I used alcohol to get out of a bad mood,” “I talked to an advisor or mentor,” and “I kept to myself, I wanted to be alone.” The remaining variables formed nine factors (Table 2). Regarding the perceived efficacy of affect regulation strategies in general, three variables were dropped because of low communalities: “I drank coffee or caffeinated beverages,” “I used alcohol to get out of a bad mood,” and “I slept or took a nap.” The remaining variables formed seven factors (Table 2).

Table 2Factor solutions of the ESEM models run with data for the frequency and efficacy of use of strategies for regulation of affect in general and sadness in particular (gf, general, frequency; ge, general, efficacy; sf, sadness, frequency; se, sadness, efficacy).

**Table:** 

	**F1 Problem-directed action, cognitive reappraisal**				**F2 Environment, physical activity**				**F3 Positive thinking**				**F4 Talking, venting**				**F5 Withdrawal, distraction**				
	**gf**	**ge**	**sf**	**se**	**gf**	**ge**	**sf**	**se**	**gf**	**ge**	**sf**	**se**	**gf**	**ge**	**sf**	**se**	**gf**	**ge**	**sf1**	**sf2**	**se**
I tried to understand my feelings by thinking and analyzing them.	**0.60**	**0.71**	**0.68**	**0.72**																	
I tried to put things in perspective.	**0.54**	**0.50**	**0.45**	**0.67**					0.48		0.47	0.37									
I made plans or a resolution to avoid such problems in the future.	**0.51**	**0.51**	**0.53**	**0.61**																	
I tried to reinterpret the situation, to find a different meaning.	**0.46**	**0.50**	**0.52**	**0.39**																	0.34
I took action to solve the problem causing my mood.	**0.46**	**0.51**	**0.38**	**0.59**										0.33							
I went for a walk in the forest, in a park, on the beach or some other natural setting.					**0.82**	**0.89**	**0.84**	**0.84**													
I went to my favorite place in nature.					**0.85**	**0.78**	**0.82**	**0.76**													
I went for a walk downtown.					**0.44**	0.45	**0.64**	**0.70**													
I went to my favorite place in an urban setting.					**0.47**	0.48	**0.56**	**0.66**													
I played sports, exercised.					**0.45**	**0.47**		0.34													
I tried to find something good in the situation.		**0.50**		0.39					**0.50**	0.43	**0.47**	**0.50**									
I tried to think about those things that are going well for me.				0.41					**0.67**	**0.75**	**0.75**	**0.70**									
I tried to be grateful for the things in my life that are going well.									**0.67**	**0.86**	**0.78**	**0.65**									
I compared myself to people who are worse off.									**0.50**	**0.38**	**0.54**	**0.66**									
I went out of my way to help someone.					0.36																
I talked to someone about my feelings.													**0.75**	**0.70**	**0.70**	**0.53**					0.35
I let my feelings out by venting or expressing them.													**0.69**	**0.56**	**0.66**	**0.49**					0.47
I talked to an advisor or mentor.	na		na		na		na		na		na		na	**0.40**	na	**0.34**	na		na	na	**0.44**
I wrote about my feelings in a diary, letter or e-mail.			na				na				na		**0.40**	**0.40**	na				na	na	
I tried to not let my feelings show, to suppress any expression.													−0.49		−**0.41**	−**0.59**	**0.50**	**0.35**			
I withdrew from or avoided the situation.																−**0.41**	**0.43**	**0.55**	**0.53**		
I daydreamed of the time when I will not have this problem.																		**0.54**	**0.38**		**0.45**
I watched TV, read a book, etc., for distraction.																		**0.45**	0.36	**0.41**	0.33
I thought about something to distract myself from my feelings.				−0.32													**0.50**	**0.47**		**0.80**	
I worked on something or stayed busy to forget my mood.																	**0.42**	**0.42**		**0.36**	
I kept to myself, I wanted to be alone.	na				na				na				na		−0.39	−**0.39**	na	**0.36**	**0.44**		
I ate something to get over my bad mood.																		**0.34**	**0.53**		
I did something fun, something I really enjoy.		0.39																			
I laughed, joked around, tried to make myself or others laugh.																					
I socialized to forget my mood.																**0.39**	0.33				
I used alcohol to get out of a bad mood.	na	na			na	na			na	na			na	na			na	na	0.35		
I treated myself to something special.																					
I prayed, put my faith in God, or did something religious.												**0.47**									
I tried to accept it as my faith, what will be, will be.

**Table:** 

	**F6 Pleasant activities, laughter**				**F7 Urban activities**				**F8 Helping others**		**F9 Faith, religion**
	**gf**	**ge**	**sf**	**se**	**gf**	**ge**	**sf**	**se**	**gf**	**sf**	**gf**
I tried to understand my feelings by thinking and analyzing them.											
I tried to put things in perspective.											
I made plans or a resolution to avoid such problems in the future.			0.38								
I tried to reinterpret the situation, to find a different meaning.											
I took action to solve the problem causing my mood.			0.35								
I went for a walk in the forest, in a park, on the beach or some other natural setting.											
I went to my favorite place in nature.											
I went for a walk downtown.						**0.56**	0.36	0.45			
I went to my favorite place in an urban setting.					0.36	**0.60**	0.43	0.54			
I played sports, exercised.				**0.39**							
I tried to find something good in the situation.										0.35	0.33
I tried to think about those things that are going well for me.											
I tried to be grateful for the things in my life that are going well.											0.33
I compared myself to people who are worse off.										0.38	
I went out of my way to help someone.									**0.39**	**0.40**	
I talked to someone about my feelings.											
I let my feelings out by venting or expressing them.											
I talked to an advisor or mentor.	na		na		na		na		na	na	na
I wrote about my feelings in a diary, letter or e-mail.			na				na			na	
I tried to not let my feelings show, to suppress any expression.				0.33							
I withdrew from or avoided the situation.				0.41							
I daydreamed of the time when I will not have this problem.									**0.43**		
I watched TV, read a book, etc., for distraction.				**0.45**	**0.46**						
I thought about something to distract myself from my feelings.		0.35		**0.64**							
I worked on something or stayed busy to forget my mood.				**0.76**							
I kept to myself, I wanted to be alone.	na				na				na		na
I ate something to get over my bad mood.	−0.44				**0.46**			**0.36**			
I did something fun, something I really enjoy.	**0.49**	**0.59**	**0.52**	**0.54**	0.32						
I laughed, joked around, tried to make myself or others laugh.	**0.48**	**0.51**	**0.44**	**0.50**					0.34		
I socialized to forget my mood.	**0.47**	**0.47**		**0.47**							
I used alcohol to get out of a bad mood.	na	na			na	na	**0.41**	**0.47**	na		na
I treated myself to something special.			**0.52**		**0.48**	**0.36**					
I prayed, put my faith in God, or did something religious.							−**0.37**			0.32	**0.72**
I tried to accept it as my faith, what will be, will be.											**0.59**

*Loadings <0.32 are not shown for clarity. In factor 5, sf1 & sf2 are two separate Withdrawal factors. Loadings in bold show the largest loadings for that item in each dataset. na: not applicable; variable deleted from the analysis due to low loadings and/or communality*.

As for frequencies of sadness regulation strategies, nine factors were retained with three variables dropped due to low communalities: “I wrote about my feelings in a diary, letter or e-mail,” “I talked to an advisor or mentor,” and “I slept or took a nap” (Table 2). As for the perceived efficacy of sadness regulation strategies, nine factors were retained with item “I slept or took a nap” dropped due to a low communality.

In all datasets, the *Environmental strategies* formed their own factor, thus being among the seven consistent factors identified in this study (F2 in Table 2). The items that loaded on it most strongly (0.78–0.89) depicted the use of natural environments for affect regulation (“I went for a walk in the forest, in a park, on the beach or some other natural setting,” “I went to my favorite place in nature”). Thus, for ANOVA, we calculated separate summary scores for *natural and urban* environmental items. In three of the four solutions, the item “I played sports, exercised” also loaded on this factor, although less strongly than the *a priori* environmental strategies (0.34–0.47). In one of the solutions, the item “I went out of my way to help someone” loaded weakly (0.36) on the environmental factor. The items on the use of urban settings for affect regulation (“I went for a walk downtown,” “I went to my favorite place in an urban setting”) loaded, in addition, on another factor whose content was less consistent in the different solutions. Other items that loaded on this *Urban activities* (F7) factor related mainly to the consumption of alcohol and food.

Other affect regulation strategies that showed a consistent pattern in the four datasets were *Problem-directed action and cognitive reappraisal* (F1), *Positive thinking* (F3), and *Talking and venting* (F4). The fifth factor, *Withdrawal and distraction*, partly overlapped with F4 (with negative loadings for the items on talking and venting) and F6 (*Pleasant activities, laughter*), representing more positive distractive strategies. In the frequency of use for sadness regulation, in contrast to other solutions, the items on the withdrawal/distraction factor formed two separate factors instead of one. In these factors, avoidance of the situation (e.g., “I withdrew from or avoided the situation”) was separate from distraction from feelings or mood (e.g., “I thought about something to distract myself from my feelings”).

Factor 8, *Helping others* was inconsistent in the two datasets where the number of factors exceeded 7, with the only common item being “I went out of my way to help someone”. Finally, one factor (F9) consisted of accepting one's *Faith and using religion* for affect regulation but that only regarded the frequency of use in general affect regulation.

### Frequency of use and perceived efficacy of environmental strategies

For frequency of use of general affect regulation, Bonferroni pairwise comparisons after ANOVA [*F*_(5.7, 2893.6)_ = 137, *p* < 0. 001] revealed that using nature as an environmental strategy was used more often than urban environments (*p* = 0.003) and with a similar frequency (= “sometimes”) as faith (*p* = 0.28) (Appendix A). All other strategies were used significantly more often than these three. For efficacy of general affect regulation, Bonferroni pairwise comparisons [*F*_(4.8, 2437.3)_ = 92, *p* < 0. 001] revealed that using nature as an environmental strategy was experienced as effective as positive thinking (*p* = 1.0) and more efficient than urban environments and withdrawal (*ps* < 0.001). The rest three strategies were experienced more effective than these (*ps* < 0.001).

For frequency of use of sadness regulation, Bonferroni pairwise comparisons after ANOVA [*F*_(4.2, 2593.6)_ = 194, *p* < 0. 001] revealed that nature as an environmental strategy was used more often than urban environments (*p* < 0. 001) but other strategies were used more often than these two. For efficacy of sadness regulation, Bonferroni pairwise comparisons [*F*_(4.2, 2622.1)_ = 136, *p* < 0. 001] revealed that using nature as an environmental strategy was experienced as effective as positive thinking (*p* = 1.0) and pleasant activities (*p* = 0.47), and more efficient than urban environments (*p* < 0. 001). Talking was experienced as the most effective strategy.

### Factor correlations

Appendices B.1–B.2 reveal that in frequency of use the environmental strategy factor is positively and significantly associated with all strategy factors except withdrawal and helping others in general affect regulation. There is a negative, nonsignificant association with urban activities in sadness regulation. The environmental strategy factor is positively and significantly associated with all other strategy factors in perceived efficacy except a negative association with urban activities in sadness regulation.

In general affect regulation (frequency of use), the environmental strategy factor correlated highest with accepting one's faith as a strategy. The second highest correlation was with the urban activities factor. In sadness regulation (frequency of use), the highest correlation of the environmental strategy was with the pleasant activities factor (i.e., doing something funny and enjoyable together or with others). The second highest correlation was with positive thinking.

Concerning the perceived efficacy of both general affect regulation and sadness regulation (Appendix B.2.), the environmental strategy factor had the highest association with positive thinking. The second highest association in general affect regulation was with withdrawal. In sadness regulation, the second highest association was with pleasant activities.

Among other strategy factors, the highest association was between pleasant activities and positive thinking and between problem-directed action/cognitive reappraisal and positive thinking.

### Relationships between affect regulation strategies and subjective well-being

In general, all strategy factors had at most modest correlations with the indicators of well-being. The overall variances explained were, nevertheless, quite high, and they were consistently greater in the models using the frequency of use of the affect regulation strategies (23–44%) than in the models using the efficacy of these strategies (14–32%). In terms of subjective well-being, it seems, thus, that using certain affect and sadness regulation strategies—when possible—predicts well-being better than the perception of their effectiveness.

For each model of affect regulation (use and efficacy, general affect and sadness), the fit indices indicated good fit with the data, as the RMSEAs ranged from 0.018–0.020, and the CFIs ranged from 0.966–0.968 (Table 3). The χ^2^ tests rejected the models, which is typical in large samples (Kline, 2015) but requires a further residual examination. The percentage of correlation residuals exceeding the |≥0.10| cutoff ranged between 1.7 and 2.6%, and the largest residuals ranged between −0.141 and 0.181, which suggests that the models did not contain any major misspecifications in absolute terms. The patterns of the residuals, however, showed that the majority of the large residuals in all four models were related to the items of emotional well-being, indicating that the conclusions related to this factor should be done with caution.

**Table 3:** ESEM model coefficients (standard error in parentheses) of the regressions of the three well-being outcomes (satisfaction with life, SWL; emotional well-being, EWB; perceived health) on the exploratory affect regulation strategy factors.

	**General frequency**			**General efficacy**			**Sadness frequency**			**Sadness efficacy**		
**Affect regulation strategy**	**SWL**	**EWB**	**Health**	**SWL**	**EWB**	**Health**	**SWL**	**EWB**	**Health**	**SWL**	**EWB**	**Health**
Problem-directed action, cognitive reappraisal	0.06 (0.07)^a^	0.04 (0.06)	0.02 (0.05)	0.12^**^ (0.04)	0.11^***^ (0.02)	0.11 (0.06)	0.14^***^ (0.03)	0.08^*^ (0.04)	−0.06 (0.05)	0.24^***^ (0.03)	0.27^***^ (0.04)	0.19^***^ (0.05)
Environment, physical activity	0.02 (0.03)	−0.01 (0.03)	0.13^*^ (0.06)	0.00 (0.04)	−0.02 (0.03)	0.11^**^ (0.03)	−0.01 (0.03)	−0.03 (0.04)	−0.08 (0.09)	0.09^*^ (0.05)	0.03 (0.02)	0.02 (0.06)
Positive thinking	0.34^***^ (0.06)	0.17^**^ (0.06)	0.09 (0.06)	0.37^***^ (0.05)	0.14^***^ (0.04)	0.17^***^ (0.03)	0.31^***^ (0.07)	0.14^***^ (0.03)	0.14^**^ (0.05)	0.08 (0.08)	0.01 (0.05)	0.04 (0.09)
Talking, venting	0.21^***^ (0.04)	0.04 (0.02)	0.16^**^ (0.06)	0.17^***^ (0.04)	0.07^*^ (0.03)	0.09 (0.05)	0.11 (0.07)	−0.01 (0.05)	0.10 (0.06)	0.12^**^ (0.04)	0.08^*^ (0.04)	0.08 (0.05)
Withdrawal, distraction^a^	−0.06 (0.06)	−0.12^*^ (0.05)	−0.04 (0.07)	−0.14^**^ (0.05)	−0.13^***^ (0.03)	−0.24^***^ (0.05)	−0.20^***^/0.02 (0.06/0.04)	−0.29^***^/−0.01 (0.04/0.03)	−0.32^***^/−0.10 (0.07/0.07)	−0.13^***^ (0.04)	−0.13^***^ (0.03)	−0.23^***^ (0.05)
Pleasant activities, laughter	0.09 (0.05)	0.14^***^ (0.04)	0.18^*^ (0.09)	0.05 (0.04)	0.00 (0.02)	0.10^*^ (0.05)	0.05 (0.08)	0.08 (0.04)	0.18^***^ (0.02)	−0.01 (0.06)	0.03 (0.04)	0.05 (0.05)
Urban activities	−0.06 (0.08)	−0.04 (0.07)	−0.09 (0.09)	−0.04 (0.06)	−0.01 (0.04)	0.04 (0.05)	0.03 (0.03)	−0.05 (0.04)	−0.12^*^ (0.06)	0.01 (0.03)	−0.10^***^ (0.02)	−0.18^***^ (0.05)
Helping others	−0.17^***^ (0.03)	−0.17^***^ (0.04)	−0.32^***^ (0.06)				−0.30^***^ (0.06)	−0.12^*^ (0.05)	−0.05 (0.08)			
Faith, religion	0.15^***^ (0.04)	0.08^**^ (0.03)	0.16^***^ (0.04)									
*R^2^*	39.5	44.4	24.1	31.8	21.0	14.6	42.0	44.3	22.8	18.1	32.3	13.8
Fit indices	*χ^2^*	value	672			827			811			965
		*df*	578			702			645			777
		*p*	0.004			0.0008			<0.0001			<0.0001
	*RMSEA*		0.018			0.019			0.02			0.02
	*CFI*		0.966			0.967			0.968			0.967

a *In the Sadness frequency data, the items in this factor loaded on two factors (estimates separated by a slash)*.

* P < 0.05,

** P < 0.01,

*** *P < 0.001*.

#### Environmental strategy

Both frequency of use and perceived efficacy for the environmental strategy factor were significantly and positively related to perceived general health. The perceived efficacy of the environmental strategy was positively related to satisfaction with life in sadness regulation. No relations of environmental strategy factor with emotional well-being within the past month were found.

#### Other strategies, positive relations

In addition to the environmental strategy, the pleasant activities strategy was the only one for which both frequency and efficacy of general affect regulation were positively related to perceived health. Moreover, frequency of the pleasant activities strategy was related to perceived health in sadness regulation and to emotional well-being in general affect regulation.

Overall, the positive thinking strategy was significantly, positively, and most consistently related to all outcomes (life satisfaction, emotional well-being and perceived health) except for self-reported efficacy of sadness regulation, in which problem-directed action and cognitive reappraisal strategy was positively related to all outcomes. In sadness regulation, also the frequency of problem-directed action and cognitive reappraisal was related to life satisfaction and emotional well-being. Moreover, the perceived efficacy of problem-directed action and cognitive reappraisal in general affect regulation was positively related to life satisfaction and emotional well-being.

The perceived efficacy of talking and venting was positively related to life satisfaction and emotional well-being both in general affect and sadness regulation. The frequency of use of talking and venting in general affect regulation was positively related to life satisfaction and perceived health. The frequency of using faith and religion in general affect regulation was positively related to all outcomes.

#### Other strategies, negative relations

In sadness regulation, both the frequency and perceived efficacy of withdrawal and distraction were consistently, significantly and negatively related to all three outcomes. In general affect regulation, the perceived efficacy of this strategy was negatively related to all three outcomes and frequency of use to emotional well-being. The frequency of helping others was negatively related to all outcomes in general affect and sadness regulation. Both the frequency and perceived efficacy of urban activities was negatively related to perceived health in sadness regulation (and also perceived efficacy to emotional well-being).

## Discussion

The purpose of this study was to examine the perceived frequency of use and efficacy of environmental strategies in relation to other strategies for regulation of affect in general and sadness more specifically. The environmental strategies of going for a walk in a natural setting or downtown or to a favorite place in nature were reported being used or being effective sometimes or moderately. On average, four strategies were used somewhat more often than the environmental strategy. These were problem-directed action, talking and venting and positive thinking and pleasant activities. Several individual activities such as using alcohol, eating, praying, or writing about one's feelings were used less often or perceived as less effective than the environmental strategy in affect regulation. As several practical obstacles may hinder the use of environments, it is noteworthy that use of natural environments was experienced as effective as positive thinking in both general and sadness regulation. It was also rated as effective as pleasant activities in sadness regulation. Concluding from these findings, it seems that the environmental strategy fits well in the “low end” among the more established strategies of affect regulation in the sense of frequency of use and perceived efficacy.

In particular, we wanted to explore whether people recognize the theoretically and empirically plausible idea of using urban and/or natural environments in affect regulation, a point of view largely neglected in earlier research on affect regulation strategies. Thus, we investigated the factorial structure of affect regulation strategies and their relationships with different indicators of subjective well-being. As was expected in Hypothesis 1, environmental regulation did form a separate category of affect regulation. Environmental items did not load on factors describing, for example, distraction (e.g., “I watched TV, read a book etc., for distraction,” “I worked on something or stayed busy to forget my mood”) or withdrawal (e.g., “I withdrew from or avoided the situation,” “I worked on something or stayed busy to forget my mood”), although they could have logically done so. Loadings on distraction or withdrawal would have shown that the choice of natural or urban environments combines with or serves other regulation strategies. However, the environmental strategy factor correlated with other strategies, suggesting potential co-occurrence or temporal sequences of using regulation strategies. As our study was cross-sectional we did not attempt to directly and fully address the likelihood that different strategies are organized in sequences. Such an effort was beyond the scope of this paper.

In general affect regulation (frequency of use), the environmental strategy factor correlated highest with accepting one's faith or being religious as a strategy reflecting the potential temporal sequence where a favorite place in an urban setting might be a church for religious activities. The second highest correlation was with urban activities which is mainly due to double loadings of the items within the environmental strategy factor and/or having, for example, a favorite pub for drinking alcohol. That urban activities were related negatively in sadness regulation may suggest that going to nature or a favorite place when sad precludes places associated with eating and alcohol. In sadness regulation (frequency of use), the highest correlation (the second highest in perceived efficacy) of the environmental strategy was with pleasant activities, that is, doing something funny and enjoyable together or with others; potentially in a natural and/or favorite place. The second highest correlation was with positive thinking, reflecting the proposed use of favorite and/or natural settings for contemplation and reflection on one's life (Kaplan and Kaplan, 1989; Korpela et al., 2001; Mayer et al., 2009). Concerning perceived efficacy of both general affect and sadness regulation, the environmental strategy had the highest associations with positive thinking. The second highest association in general affect regulation was with withdrawal reminding that withdrawal is most often from somewhere to somewhere else, potentially to a favorite place permitting or even promoting positive thinking.

However, as the emergence of an independent environmental strategy factor might be due to particular sample characteristics and to the synonymic wordings of items, a more encouraging support for the concept of environmental means of affect regulation is the fact that it was also positively associated with well-being, supporting Hypothesis 2. Namely, the higher the frequency of use and the perceived efficacy of the Environmental strategy were for general affect regulation, the healthier people felt. The more effective the Environmental strategy was perceived for sadness regulation the more satisfied with their life the respondents were. These results are in accordance with the idea of restorative (mostly natural) environments providing benefits for well-being (Bowler et al., 2010; Hartig et al., 2014; Kondo et al., 2018). Further, in the regression model, not all strategies had positive associations with perceived health which highlights the importance of including environmental strategies in affect regulation and coping repertoires. We regard these results as noteworthy findings in a situation where preceding studies have not included environmental strategies in their models.

No relations of the environmental strategies with emotional well-being within the past month (e.g., nervousness, depressiveness, calmness and happiness) were found. This is contrary to what one would expect when considering that natural environments support most reliably happiness (MacKerron and Mourato, 2013) and positive emotional and mood change (improvement in the feelings of anxiety, anger, fatigue, energy and sadness) (Bowler et al., 2010) and studies of motives for outdoor recreation have commonly referred to escape from stressors as well as a search for positive experiences (Knopf, 1987). However, previous studies have not included several affect regulation strategies as predictors of life satisfaction or emotional well-being in their statistical models. These inconsistent results may also be due to the co-presence of items referring to both natural and built environments in the environmental strategy factor. Earlier studies have indicated that people select urban places as their favorite places, and we wanted to include both urban and natural settings in our strategy factor although urban favorite places seem to evoke somewhat less intensive positive feelings than favorite natural settings (Korpela et al., 2010; cf. Staats et al., 2016). The use of urban favorite places loaded also on the factor including eating and drinking alcohol. As this factor was consistently negatively or not at all associated with emotional well-being (and perceived health), particularly in sadness regulation, this may have lowered the positive association of the Environmental strategy factor with emotional well-being.

Regarding other affect regulation strategies, the positive thinking strategy was significantly, positively, and most consistently related to all outcomes (life satisfaction, emotional well-being and perceived health) except for self-reported efficacy of sadness regulation in which the problem-directed action and cognitive reappraisal strategy was positively related to all outcomes. In sadness regulation, also the frequency of the problem-directed action and cognitive reappraisal strategy was related to life satisfaction and emotional well-being. The perceived efficacy of the problem-directed action and cognitive reappraisal strategy in general affect regulation was positively related to life satisfaction and emotional well-being. These results conform with earlier studies that have found cognitive reappraisal to correlate positively with positive affect and life satisfaction (Haga et al., 2009), and higher levels of psychological well-being (Park and Adler, 2003).

The positive link between the talking and venting factor and the well-being outcomes conforms with previous findings of a link between approach-oriented coping strategies such as social approach, reflected in the talking and venting factor, and positive affect, psychological, and physical health in stressful circumstances (Taylor and Stanton, 2007). In accordance with the current results, there is previous evidence of religious coping strategies being associated with positive outcomes to stressful events such as more stress-related growth, positive affect, and higher self-esteem and less depression, anxiety, or distress (Ano and Vasconcelles, 2005). On the other hand, frequent use and efficacy of the withdrawal and distraction strategy in sadness regulation were negatively associated with all well-being measures. In general effect regulation, perceived efficacy of this strategy was negatively related to all three outcomes and frequency of use to emotional well-being. The results resemble earlier results showing that the reliance on the avoidance coping strategies is associated with higher levels of anxiety among students (Park and Adler, 2003) and depressive symptoms in the late-middle-aged people (Holahan et al., 2005). Surprisingly, the frequency of helping others was negatively related to all outcomes in general affect and sadness regulation. This may be due to the fact that daydreaming and comparisons to other people also loaded on this factor. Daydreaming as a form of withdrawal and comparing oneself to others perceived to be healthier or better off in some way may worsen well-being.

### Limitations and future directions

Due to the relatively small sample sizes it was not possible to extensively examine the relationships between affect regulation strategies and subjective well-being by country. Instead, country-wise differences were controlled for in our analyses. Similarly, we did not examine gender differences in coping strategies, though some research indicates they are common (Tamres et al., 2002). These are matters for future research. Since the majority of participants were students, any generalizations to the general public should be made with caution. It should also be noted that regressions performed with cross-sectional data cannot reveal the causal direction of associations, so any such conclusions based on them in that regard are speculative. It is possible, for example, that people who perceive their health as good are prone to perceive favorite environments as effective in regulating their feelings, not the other way around. We have left the question of temporal sequences in the use of affect regulation strategies for future studies; in the area of emotion regulation and coping it is an important but still under-researched area (Carver and Connor-Smith, 2010; Naragon-Gainey et al., 2017).

It is also up to future studies to take a closer look at the relative use and effectiveness of different affect regulation strategies and cultural differences among them. The environmental strategy deserves further attention in the field of coping and affect regulation studies because its effectiveness in stress reduction has already led to various practical applications, such as “favorite place prescriptions” (Korpela and Ylén, 2009), “green prescriptions” (Marselle et al., 2013), and “health walks” (Bird, 2004). These prescriptions are physical activity or stress recovery recommendations from health care practitioners to the general population or special groups, such as people suffering from depression and inactive people who are insensitive to health education that emphasizes physical activity for the sake of physical fitness alone. Comparative studies of several affect regulation strategies or “prescriptions” are needed. Favorite places serving affect regulation may not be limited to the local, residential scale only (Bijker and Sijtsma, 2017), which indicates a need for research at different spatial levels.

The present study included only the general affect regulation and sadness. In future research, affect regulation could be studied with relation to several specific emotions. For example, which strategies are used most often and perceived to be the most effective in the regulation of anger? It has been found that adolescents rely on leaving the scene to socially manage their anger (Keiley and Seery, 2001). Leaving to where, could future researchers ask.

## Conclusion

Environmental strategies of affect regulation refer to the use of specific places or socio-physical settings in the service of affect regulation, such as visiting a nearby favorite place in a natural or urban setting to calm down. These strategies have gone largely unnoticed in research on affect regulation. In this study, such strategies formed a separate factor of affect regulation and had positive relations to perceived health in general affect regulation and to life satisfaction in regulating sadness. Not all of the more established regulation strategies had positive associations with perceived health which highlights the importance of including environmental strategies in future affect regulation research and also in coping research.

## Author contributions

KK: designed the study; TP: analyzed the results; KK, TP, VR, TH, HS, and MM: made a substantial, intellectual contribution to the work, all the listed authors gathered the data in their respective countries and approved the work for publication.

### Conflict of interest statement

The authors declare that the research was conducted in the absence of any commercial or financial relationships that could be construed as a potential conflict of interest.
