# Supplementary material for: Environmental Strategies of Affect Regulation and Their Associations With Subjective Well-Being
Source: Front Psychol. 2018 Apr 18;9:562. doi: 10.3389/fpsyg.2018.00562 (PMC5915835; doi:10.3389/fpsyg.2018.00562)
Supplement: Supplementary file 1 [file Table1.docx]

Appendix A. Means, standard deviations (*SD*s, in brackets) and factor mean summary scores (*s.e.,* in brackets) for the ANOVA of the affect regulation strategy items.

| Items | Factor | gf (n=565) | ge (n=565) | sf (n=626) | se (n=626) |
| --- | --- | --- | --- | --- | --- |
| I tried to understand my feelings by thinking and analyzing them. | Problem-directed action, cognitive reappraisal (PD)_a,b_ | 4.32 (1.45) | 3.43 (1.49) | 4.32 (1.42) | 3.25 (1.47) |
| I tried to put things in perspective. | PD_a,b_ | 3.38 (1.34) | 3.14 (1.36) | 3.55 (1.35) | 3.22 (1.40) |
| I made plans or a resolution to avoid such problems in the future. | PD_a,b_ | 3.46 (1.49) | 3.02 (1.52) | 3.34 (1.47) | 2.78 (1.49) |
| I tried to reinterpret the situation, to find a different meaning. | PD_a,b_ | 2.85 (1.46) | 2.88 (1.47) | 2.86 (1.52) | 2.76 (1.48) |
| I took action to solve the problem causing my mood. | PD_a,b_ | 3.42 (1.38) | 3.57 (1.48) | 3.40 (1.46) | 3.40 (1.49) |
| **Mean summary score (*s.e.*)** |  | **3.5 (.04)** | **3.2 (.05)** | **3.5 (.04)** | **3.1 (.05)** |
| I went for a walk in the forest, in a park, on the beach or some other natural setting. | Environmental, nature (NEnv)_a,b,c_ | 2.35 (1.66) | 2.98 (1.71) | 2.44 (1.64) | 2.94 (1.64) |
| I went to my favorite place in nature. | NEnv_a,b_ | 2.16 (1.54) | 2.69 (1.65) | 2.07 (1.53) | 2.71 (1.64) |
| **Mean summary score (*s.e.*)** |  | **2.3 (.06)** | **2.8 (.07)** | **2.3 (.06)** | **2.8 (.06)** |
| I went for a walk downtown. | Environmental, urban (UEnv)_a,b,c_ | 2.27 (1.53) | 2.19 (1.49) | 2.20 (1.57) | 2.11 (1.47) |
| I went to my favorite place in an urban setting. | UEnv_a,b_ | 1.75 (1.46) | 2.05 (1.51) | 1.58 (1.38) | 1.89 (1.46) |
| **Mean summary score (*s.e.*)** |  | **2.0 (.06)** | **2.1 (.06)** | **1.9 (.05)** | **2.0 (.05)** |
| I played sports, exercised. |  | 2.60 (1.75) | 3.03 (1.76) | 2.57 (1.76) | 2.98 (1.81) |
| I tried to find something good in the situation. | Positive thinking (PT)_a,b_ | 3.22 (1.43) | 3.06 (1.43) | 3.03 (1.45) | 2.86 (1.43) |
| I tried to think about those things that are going well for me. | PT_a,b_ | 3.41 (1.40) | 3.18 (1.45) | 3.37 (1.37) | 3.00 (1.36) |
| I tried to be grateful for the things in my life that are going well. | PT_a,b_ | 3.79 (1.53) | 3.22 (1.58) | 3.57 (1.49) | 3.04 (1.48) |
| I compared myself to people who are worse off. | PT_a,b_ | 2.38 (1.52) | 2.20 (1.54) | 2.52 (1.53) | 2.17 (1.52) |
| **Mean summary score (*s.e.*)** |  | **3.2 (.05)** | **2.9 (.05)** | **3.1 (.05)** | **2.8 (.05)** |
| I went out of my way to help someone. | Helping others | 2.16 (1.45) | 2.56 (1.54) | 1.84 (1.33) | 2.33 (1.53) |
| I talked to someone about my feelings. | Talking, venting (T)_a,b_ | 3.48 (1.60) | 3.73 (1.60) | 3.50 (1.63) | 3.87 (1.52) |
| I let my feelings out by venting or expressing them. | T_a,b_ | 2.75 (1.44) | 3.02 (1.52) | 2.85 (1.53) | 3.14 (1.56) |
| **Mean summary score (*s.e.*)** |  | **3.1 (.06)** | **3.4 (.06)** | **3.2 (.06)** | **3.5 (.05)** |
| I talked to an advisor or mentor. | T | 1.24 (1.45) | 2.08 (1.74) | 1.15 (1.41) | 2.16 (1.82) |
| I wrote about my feelings in a diary, letter or e-mail. | T | 1.67 (1.71) | 2.14 (1.85) | 1.89 (1.75) | 2.36 (1.85) |
| I tried to not let my feelings show, to suppress any expression. | T | 2.83 (1.48) | 1.68 (1.41) | 2.91 (1.52) | 1.54 (1.36) |
| I withdrew from or avoided the situation. | Withdrawal, distraction (W)_a,b_ | 2.42 (1.36) | 1.86 (1.44) | 2.58 (1.44) | 1.74 (1.41) |
| I daydreamed of the time when I will not have this problem. | W | 2.71 (1.78) | 1.83 (1.61) | 2.89 (1.80) | 1.84 (1.55) |
| I watched TV, read a book, etc., for distraction. | W | 3.47 (1.44) | 2.93 (1.46) | 3.42 (1.49) | 2.80 (1.47) |
| I thought about something to distract myself from my feelings. | W_a,b_ | 2.84 (1.37) | 2.60 (1.40) | 3.03 (1.42) | 2.62 (1.43) |
| I worked on something or stayed busy to forget my mood. | W_a,b_ | 3.07 (1.33) | 2.99 (1.41) | 3.08 (1.43) | 2.88 (1.45) |
| **Mean summary score (*s.e.*)** |  | **2.8 (.04)** | **2.5 (.05)** | **-** | **-** |
| I kept to myself, I wanted to be alone. | W | 2.88 (1.47) | 2.15 (1.51) | 2.94 (1.60) | 1.97 (1.51) |
| I ate something to get over my bad mood. | W | 2.21 (1.60) | 1.38 (1.37) | 2.32 (1.68) | 1.36 (1.36) |
| I did something fun, something I really enjoy. | Pleasant activities, laughter (P) _a,b_ | 3.48 (1.29) | 3.69 (1.40) | 2.97 (1.42) | 3.38 (1.47) |
| I laughed, joked around, tried to make myself or others laugh. | P_a,b_ | 2.85 (1.61) | 2.93 (1.59) | 2.43 (1.60) | 2.57 (1.57) |
| I socialized to forget my mood. | P_a_ | 2.48 (1.43) | 2.94 (1.49) | 2.52 (1.45) | 2.92 (1.52) |
| **Mean summary score (*s.e.*)** |  | **2.9 (.05)** | **3.2 (.05)** | **2.7 (.05)** | **3.0 (.05)** |
| I used alcohol to get out of a bad mood. | Urban activities (U) | 1.05 (1.37) | 1.06 (1.45) | 1.36 (1.54) | 1.27 (1.52) |
| I treated myself to something special. | U | 2.53 (1.35) | 2.63 (1.48) | 2.48 (1.40) | 2.45 (1.43) |
| I prayed, put my faith in God, or did something religious. | Faith, religion (F)_a_ | 1.76 (2.05) | 1.84 (2.07) | 1.37 (1.73) | 1.49 (1.78) |
| I tried to accept it as my faith, what will be, will be. | F_a_ | 2.31 (1.56) | 2.11 (1.62) | 2.38 (1.61) | 2.01 (1.61) |
| **Mean summary score (*s.e.*)** |  | **2.0 (.07)** | **-** | **-** | **-** |

*Note.* gf = general, frequency; ge = general, efficacy; sf = sadness, frequency; se = sadness, efficacy; a = items used for the factor mean summary score (in ANOVA), general affect regulation, b = items used for the factor mean summary score (in ANOVA), sadness regulation, c = for the Environmental strategy, separate summary scores for nature and urban items were calculated.
