# Supplementary material for: Environmental Strategies of Affect Regulation and Their Associations With Subjective Well-Being
Source: Front Psychol. 2018 Apr 18;9:562. doi: 10.3389/fpsyg.2018.00562 (PMC5915835; doi:10.3389/fpsyg.2018.00562)
Supplement: Supplementary file 2 [file Table2.docx]

Appendix B.1. Estimated bivariate correlations between the latent factors in the *frequency of using* general affect regulation strategies (upper triangle) and sadness strategies (lower triangle).

|  |  | F1 | F2 | F3 | F4 | F5* | F6 | F7 | F8 | F9 | SWL | EWB | Health |
| --- | --- | --- | --- | --- | --- | --- | --- | --- | --- | --- | --- | --- | --- |
| F1 | Problem-directed action, cognitive reappraisal |  | .14 | **.24** | **.26** | -.01 | .11 | .03 | .09 | **.24** | **.26** | .20 | .13 |
| F2 | Environment, physical activity | **.25** |  | **.18** | **.18** | .05 | **.18** | **.24** | .15 | **.26** | **.16** | .06 | **.18** |
| F3 | Positive thinking | **.32** | **.22** |  | **.20** | -.01 | **.37** | .11 | .14 | **.10** | **.49** | **.39** | **.17** |
| F4 | Talking, venting | .14 | **.14** | **.18** |  | .09 | **.16** | **.20** | .05 | .12 | **.35** | **.15** | **.21** |
| F5* | Withdrawal, distraction | -.03 / **.10** | -.04 / **.11** | **-.16 / .28** | **-.09** / -.02 | .15** | -.02 | **.32** | **.18** | .07 | -.10 | **-.30** | -.09 |
| F6 | Pleasant activities, laughter | **.21** | **.28** | **.41** | **.21** | **-.13 / .21** |  | **.18** | -.06 | .09 | **.32** | **.42** | **.28** |
| F7 | Urban activities | -.07 | -.12 | .00 | .06 | -.06 / .11 | **.16** |  | **.19** | **.22** | .03 | -.09 | -.02 |
| F8 | Helping others | .17 | **.17** | .18 | .09 | **.16 / .27** | **.14** | **.20** |  | **.19** | -.11 | **-.31** | **-.28** |
| F9 | Faith, religion | na | na | na | na | na | na | na | na |  | **.21** | **.12** | **.16** |
| SWL | Satisfaction with life | **.25** | .09 | **.44** | **.21** | **-.37** / .02 | **.26** | -.01 | -.01 | **-.26** |  | **.68** | **.47** |
| EWB | Emotional well-being | **.20** | .06 | **.34** | .08 | **-.55** / -.06 | **.24** | -.08 | -.08 | **-.22** | **.66** |  | **.46** |
| Health | General health | .01 | .01 | **.20** | **.16** | **-.38** / -.12 | **.21** | -.08 | -.08 | **-.12** | **.38** | **.46** |  |

*Note.* na = not applicable; factor not present in the solution. Correlation coefficients in bold: *p*<.05

* In the frequency of sadness regulation strategies, two factors (F5_1, F5_2) loaded on the items that formed F5 in other datasets

** The correlation between F5_1 and F5_2 in the sadness regulation data
