# Supplementary material for: Environmental Strategies of Affect Regulation and Their Associations With Subjective Well-Being
Source: Front Psychol. 2018 Apr 18;9:562. doi: 10.3389/fpsyg.2018.00562 (PMC5915835; doi:10.3389/fpsyg.2018.00562)
Supplement: Supplementary file 3 [file Table3.docx]

Appendix B.2. Estimated bivariate correlations between the latent factors in the *perceived efficacy* of using general affect regulation strategies (upper triangle) and sadness strategies (lower triangle).

|  |  | F1 | F2 | F3 | F4 | F5 | F6 | F7 | SWL | EWB | Health |
| --- | --- | --- | --- | --- | --- | --- | --- | --- | --- | --- | --- |
| F1 | Problem-directed action, cognitive reappraisal |  | **.27** | **.45** | .16 | **.27** | .03 | **.19** | **.32** | **.28** | **.17** |
| F2 | Environment, physical activity | **.23** |  | **.24** | **.18** | **.23** | **.14** | **.22** | **.14** | .05 | **.16** |
| F3 | Positive thinking | **.27** | **.30** |  | **.17** | **.27** | **.20** | **.28** | **.48** | **.31** | **.23** |
| F4 | Talking, venting | **.27** | **.18** | .02 |  | -.02 | **.18** | .04 | **.30** | **.21** | **.18** |
| F5 | Withdrawal, distraction | .15 | **.22** | **.18** | **-.13** |  | .01 | **.30** | -.02 | **-.14** | **-.13** |
| F6 | Pleasant activities, laughter | **.30** | **.25** | **.45** | .05 | **.34** |  | **.20** | **.17** | .07 | **.18** |
| F7 | Urban activities | .07 | **-.09** | **.15** | -.08 | **.16** | **.29** |  | .07 | .01 | .08 |
| SWL | Satisfaction with life | **.47** | **.16** | .12 | **.31** | **-.16** | **.09** | **-.16** |  | **.67** | **.47** |
| EWB | Emotional well-being | **.34** | **.19** | **.17** | **.26** | **-.09** | .10 | -.01 | **.66** |  | **.45** |
| Health | General health | **.20** | .07 | .06 | **.18** | **-.21** | .01 | **-.19** | **.46** | **.38** |  |

*Note.* Correlation coefficients in bold: *p*<.05
